# Pneumatosis intestinalis after gefitinib therapy for pulmonary adenocarcinoma: a case report

**DOI:** 10.1186/s12957-016-0926-1

**Published:** 2016-06-29

**Authors:** Ai Maeda, Masao Nakata, Katsuhiko Shimizu, Takuro Yukawa, Shinsuke Saisho, Riki Okita

**Affiliations:** 1 Department of General Thoracic Surgery, Kawasaki Medical School, 577 Matsushima, Kurashiki, Okayama 701-0192 Japan; 2 Department of General Surgery, Kawasaki Medical School, Okayama, Okayama 700-8505 Japan

## Abstract

**Background:**

Pneumatosis intestinalis (PI) is defined as the presence of gas in the bowel wall and is a relatively rare finding. PI has been associated with various pathological conditions and medications. Although several chemotherapeutic agents and molecular targeted therapy agents are reported to be associated with PI, there have been few reports describing the association between the anti-epidermal growth factor receptor agent gefitinib, a tyrosine kinase inhibitor (TKI), and PI. The present report describes a case of PI secondary to gefitinib therapy.

**Case presentation:**

An 80-year-old woman who had been diagnosed with recurrent lung adenocarcinoma presented with remarkable appetite loss, abdominal distension, and constipation after starting gefitinib therapy. A computed tomography (CT) scan of the abdomen revealed PI extending from the small intestine to the rectum. The patient was managed conservatively, and gefitinib therapy was discontinued. Subsequently, the symptoms improved and a follow-up abdominal X-ray showed a reduction in intramural air. After gefitinib was restarted, PI occurred three more times.

**Conclusions:**

Although PI is extremely rare, physicians should be aware of the risk of PI in patients undergoing gefitinib therapy.

## Background

Pneumatosis intestinalis (PI) is relatively rare finding characterized by the presence of gas within the intestinal wall. This can be caused by bowel ischemia, mechanical trauma, inflammatory disease, autoimmune disease, intestinal neoplasm, obstructive pulmonary disease, thromboembolism, or several medications [1–3]. Recently, molecular-targeted drugs have been shown to cause gastrointestinal toxicity, including PI perforation, enteritis, and fistula formation [1, 4–6]. Anti-epidermal growth factor receptors such as cetuximab and erlotinib have been reported to be associated with PI; however, only a few cases have been reported in association with gefitinib treatment [1, 7–9]. This is a report of PI associated with gefitinib therapy.

## Case presentation

**Fig. 1:**
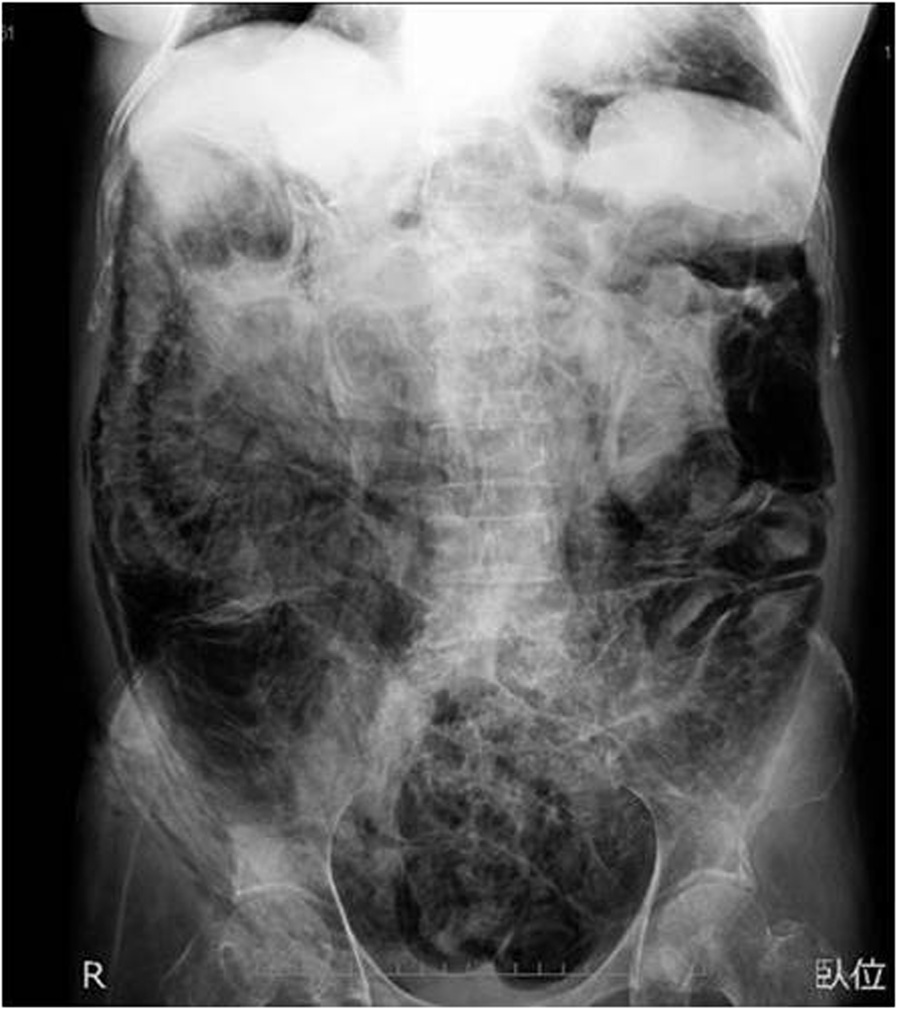
Abdominal X-ray performed before treatment revealed intestinal dilation and intraluminal gas

**Fig. 2:**
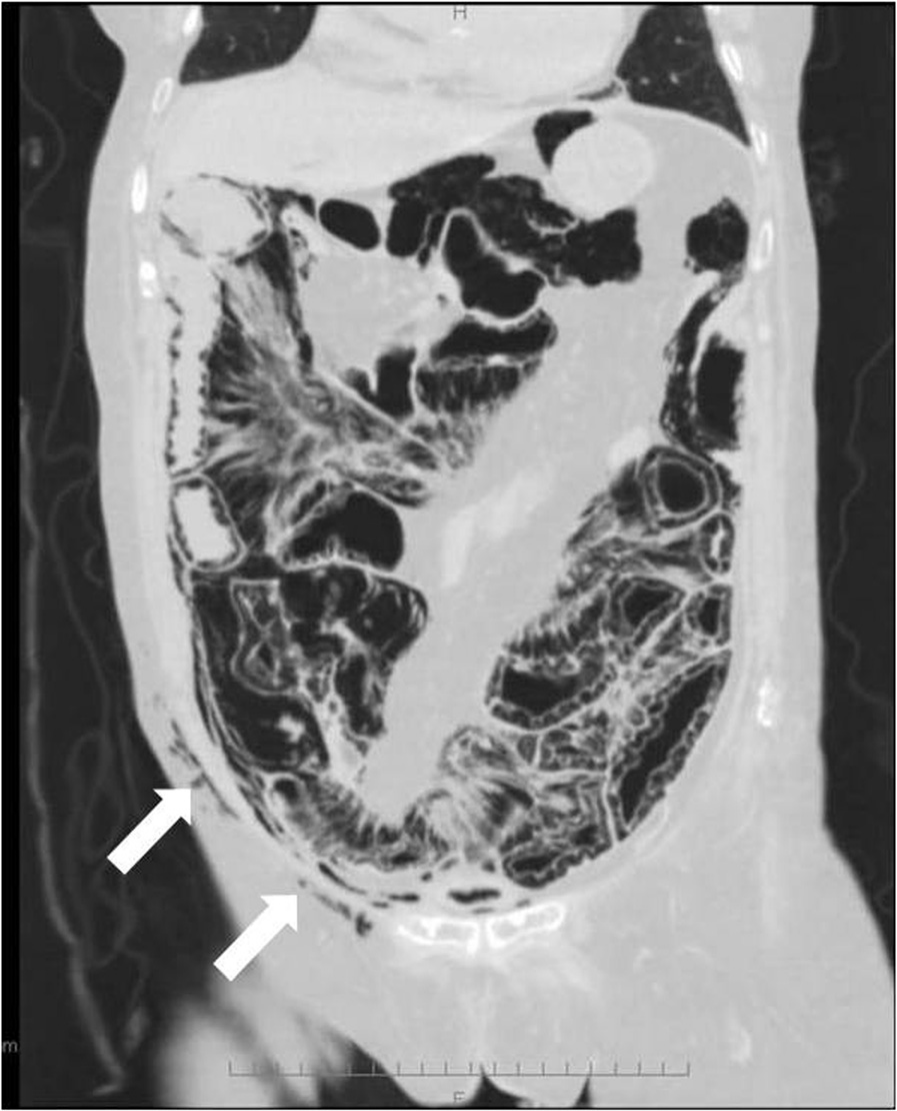
A coronal CT scan of the abdomen showed severe PI and subcutaneous emphysema (*white arrow*). Gas tracking wall is visualized parallel to the intestinal mucosa

An 80-year-old female patient with recurrent lung cancer was admitted to our hospital because of anorexia, abdominal distension, and constipation. She had been diagnosed with recurrent left lung cancer with multiple bone metastases. She had been treated with oral gefitinib for about 6 weeks, which appeared to stabilize the disease. Her past medical history included rheumatoid arthritis (RA), cor pulmonale, bronchial asthma, and hypothyroidism, which were all under medical control. She was receiving prednisolone (PSL, 10 mg/day) for RA treatment. An abdominal X-ray showed an abnormal intestinal gas shadow (Fig. 1), and computed tomography (CT) revealed intestinal dilation with diffuse thickening of practically the entire intestinal wall, intramural gas, and intraperitoneal free air, indicating PI (Fig. 2). We selected conservative treatment with I.V. antibiotics because the increase in inflammatory parameters in the blood samples was mild (white blood cell (WBC), 8430/μL; C-reactive protein (CRP), 7.11 mg/dL; pH, 7.337; and lactate, 0.87 mEq/L) and no signs of sepsis, free gas near the portal vein, or bowel perforation were detected. We discontinued the oral intake of all drugs including gefitinib.

**Fig. 3:**
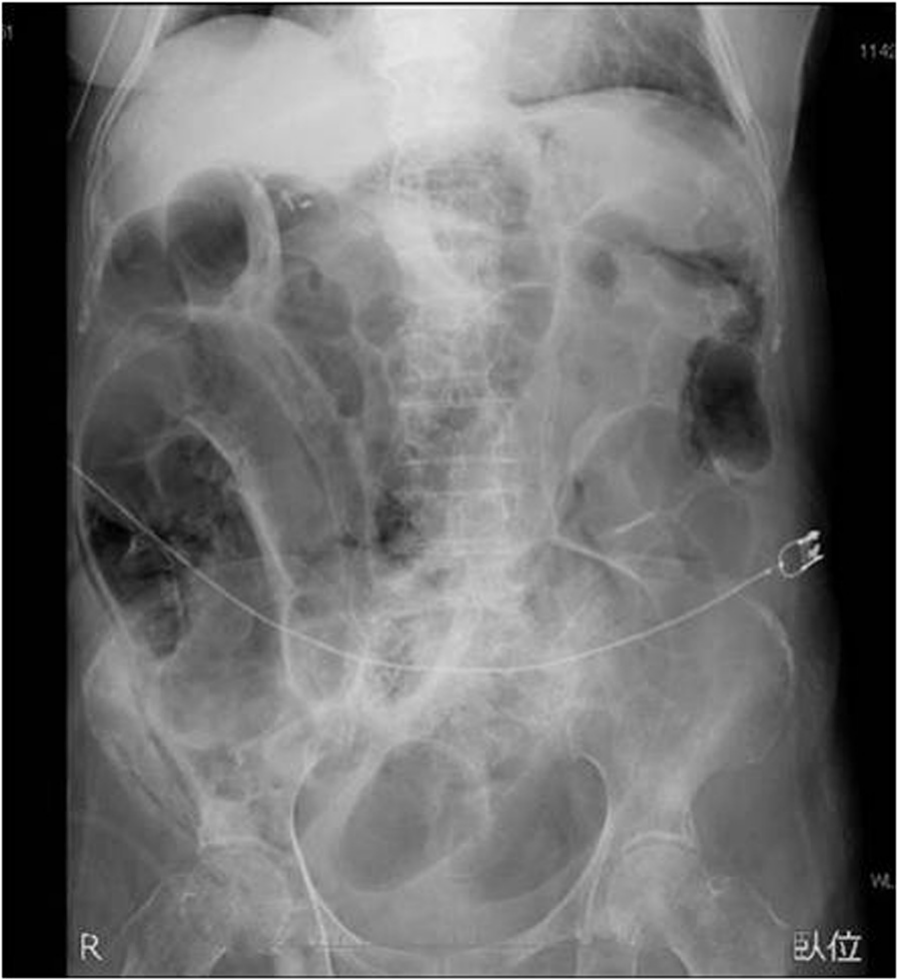
Abdominal X-ray performed after treatment revealed decreased intraluminal gas

We speculated that this patient’s PI was induced by PSL therapy; however, it was not possible to decrease the amount of PSL because of RA. The symptoms gradually improved, and a follow-up abdominal X-ray revealed decreased intramural air (Fig. 3). Considering the good response of lung cancer to gefitinib, the drug was restarted; however, within 2 weeks of gefitinib re-initiation, the patient redeveloped marked abdominal distention, and extensive pneumatosis led to drug cessation.

The same episode occurred three times during gefitinib therapy. We discontinued the administration of gefitinib after PI recurred the third time and the patient did not experience further episodes after discontinuing the drug.

### Discussion

PI is a condition of extraluminal gas predominantly localized to the submucosal and subserosal planes of the intestine, but it can also be found in the muscularis propria.

Multiple theories exist for the cause of PI onset. Three different possibilities for the source of gas within the intestinal wall have been considered, including intraluminal gas, pulmonary gas, and gas produced by bacteria. Mucosal damage, increased intraluminal pressure, or both contribute to PI. Mucosal damage may result from inflammation, a defect in the gut barrier function, and steroid- or molecular-targeted therapy. Many PI-associated conditions have been described with immunosuppressive therapy as a possible causative factor. It has been suggested that long-term administration of corticosteroids induces atrophy of the intestinal mucosa, which sometimes results in a mucosal defect and subsequent translocation of gas into the submucosal layer [10–12]. We initially speculated that PSL treatment was the cause of PI. However, considering the fact PI recurred after the initiation of gefitinib therapy, we concluded that gefitinib therapy led to PI, although the underlying mechanism is not well understood.

Several recent reports have suggested that PI is associated with molecular targeted therapy agents such as vascular endothelial growth factor (VEGF) inhibitors, tyrosine kinase inhibitors (TKI), mammalian target of rapamycin (mTOR) inhibitors, and immune modulators [1]. Molecular-targeted therapies can be beneficial for treating cancers but can often induce severe bowel toxicity. This effect is particularly true with VEGF inhibitors such as bevacizumab, which has been shown to increase the risk of perforation. It has been hypothesized that the decreased blood supply caused by angiogenesis inhibition may reduce the capillary density of intestinal villi, possibly causing abdominal hypoxia and microperforation and allowing air to infiltrate the bowel wall [1, 5].

In general, gefitinib has a good toxicity profile among the targeted molecular therapy agents. However, some patients develop specific and severe toxicities because these molecular targets also affect normal cells. Although gefitinib is generally well tolerated, its most commonly reported toxicities concern the gastrointestinal tract (diarrhea, nausea, and vomiting) and skin (rash, acne, dry skin, and pruritus). Severe gastrointestinal toxicity caused by gefitinib is uncommon, and only 1 % of patients receiving gefitinib therapy develop grade 3 or 4 diarrhea [9, 13]. To the best of our knowledge, only a few studies, including the current case report, have reported the association between PI and gefitinib [8, 9].

Most cases of PI are asymptomatic or mild; however, in the present case, the patient’s symptoms were anorexia and abdominal distension. Patients with PI most frequently present with vomiting, abdominal distension, and abdominal discomfort [14]. These diagnoses are made radiographically and not by symptoms. Conservative management is preferable; however, in cases involving elevated CRP or WBC levels as well as signs of sepsis, bowel perforation, or free gas near the portal vein, immediate surgery is indicated [13–16]. In this case, the patient’s CRP levels remained at higher than normal levels on account of RA. Because we did not observe intestinal perforation or an increase in WBC levels, we selected conservative treatment.

## Conclusions

This is a rare case report of PI caused by gefitinib. The symptoms of PI appear to be minor; however, this condition has the potential to lead to intestinal perforation. Although this complication is extremely rare, physicians should be aware of the risk of PI in patients undergoing gefitinib therapy.

## Abbreviations

CT, computed tomography; mTOR, mammalian target of rapamycin; PI, pneumatosis intestinalis; PSL, prednisolone; RA, rheumatoid arthritis; TKI, tyrosine kinase inhibitor; VEGF, vascular endothelial growth factor
